# Ebola, Zika and the International Health Regulations – implications for Port Health Preparedness

**DOI:** 10.1186/s12992-016-0173-9

**Published:** 2016-11-21

**Authors:** R. W. Glynn, M. Boland, On behalf of the HSE Port Health Groups, Ireland

**Affiliations:** Department of Public Health East, Health Service Executive, Dr. Steevens’ Hospital, Steevens’ Lane, Dublin, 8 Ireland

**Keywords:** ‘International health regulations”, IHR, Ebola, EVD, “Port Health”, Zika

## Abstract

**Background:**

The outbreak of Ebola Virus Disease in West Africa in 2014-2015 was unprecedented in terms of its scale and consequence.  This, together with the emergence of Zika virus as a Public Health Emergency of International Concern in 2016, has again highlighted the potential for disease to spread across international borders and provided an impetus for countries to review their Port Health preparedness. This report reviews the legislative framework and actions taken under this framework in advancing and improving Port Health preparedness in Ireland, in response to the declaration of the Public Health Emergency of International Concern for Ebola Virus Disease in August 2014.

**Findings:**

Infectious disease Shipping and Aircraft Regulations were brought into force in Ireland in 2008 and 2009, respectively. Preparatory actions taken under these and the International Health Regulations necessitated significant levels of cross disciplinary working with other organisations, both within and beyond traditional healthcare settings. Information packs on Ebola Virus Disease were prepared and distributed to airports, airlines, port authorities and shipping agents, and practical exercises were held at relevant sites. Agreements were put in place for contact tracing of passenger and crew on affected conveyances and protocols were established for the management of Medical Declarations of Health from ships coming from West Africa.

**Conclusions:**

The outbreak of Ebola Virus Disease in West Africa resulted in significant strengthening of Ireland’s Port Health preparedness, while also highlighting the extent to which preparedness requires ongoing and sustained commitment from all stakeholders, both nationally and internationally, in ensuring that countries are ready when the next threat presents at their borders.

## Introduction

Over the last few decades, a number of emerging infectious diseases have taken the global community by surprise including HIV, SARs, H1N1, MERS-CoV and Ebola Virus Disease (EVD)[1]. The outbreak of the latter in West Africa in 2014-2015 was unprecedented in terms of its scale and consequence, and was responsible for the deaths of more than 11,000 people.  The emergence of Zika virus in Latin America and the Caribbean in 2015-2016, meanwhile, has demonstrated that weaknesses in public health preparedness and response capabilities were neither unique to West Africa nor to EVD, and reiterate the need for renewed focus on these aspects of global health protection [2].

It was with scenarios like EVD and Zika in mind that the International Health Regulations (IHR) were updated and brought into force in 2007. Their stated purpose and scope was ‘to prevent, protect against, control and provide a public health response to the international spread of disease in ways that are commensurate with and restricted to public health risks, and which avoid unnecessary interference with international traffic and trade’. Although the IHR are aimed at several areas of public health security they can be broadly summarized into two main areas; 

- Urgent actions to be taken with respect to acutely arising risks to public health and
- Strengthening of national systems and infrastructure (core capacities)

While Ireland has no direct flights from the affected countries in West Africa, a significant number of travelers who leave that area arrive in Ireland as their final destination. At least 11,000 people travelled from the region to the UK alone between September and December 2014, for example, and it can be expected that a reasonable number travelled onwards to Ireland. In addition, Ireland also had to contend with significant numbers of returning humanitarian aid workers from the region; between December 2014 and November 2015, 83 of these workers returned to Ireland from their posts in West Africa.

Although Ireland has no direct airline traffic from West Africa, a number of ships do travel directly from there with cargo and hence there was heightened concern that a crew member may arrive with symptoms. Rusal Aughinish in County Limerick, for example, is Europe’s largest bauxite refinery. Bauxite is used to make aluminium and Guinea was the sixth largest global producer in 2012 [3]. Ireland is a key export market for Guinea, with over $90 million worth of bauxite imported from there annually. The shipping traffic to Ireland from West Africa was therefore significant, with at least 44 ships arriving at Irish ports between August 2014 and May 2015, having previously called at ports in the affected countries.

This paper aims to discuss the multisectoral work which was involved in advancing and improving Port Health preparedness in Ireland, in light of the IHR and, in particular, in light of the outbreak of EVD in West Africa in 2014.

### Legislative background in Ireland

With the IHR’s requirement to strengthen core capacities in mind, the Infectious Disease (ID) Shipping Regulations and the Infectious Disease Aircraft Regulations were set down in Ireland in 2008 and 2009. While both sets of Regulations outline a schedule of infectious diseases to which they apply, this schedule reflects a key feature of the IHR in that it also provides for the inclusion of unspecified, novel threats of international importance, should they arise (Table 1).

**Table 1:** Schedule of Infectious Diseases as set out in Aircraft and Shipping Regulations, Ireland

Cholera.
Pneumonic Plague.
Yellow fever.
Viral haemorrhagic fevers.
West Nile fever.
Smallpox.
Poliomyelitis due to wild type poliovirus.
Human influenza caused by a new sub-type.
Severe acute respiratory syndrome (SARS)
Dengue fever, Rift Valley fever and meningococcal disease and any other infectious disease in respect of a person on board an aircraft originating in, coming from, or having passed through an area where any of those infectious diseases are of special national or regional concern.
Any other infectious disease which is of public health concern and of international importance

The ID Shipping Regulations identify Ireland’s five designated ports and set out the responsibilities of ships’ masters and port health authorities at these ports. The legislation requires ships’ masters to complete a Maritime Declaration of Health (MDH) where requested to do so by local port health authorities. It also provides for the inspection and detention of ships and for the removal of arrived ships to a mooring station. Furthermore, it mandates the provision of a Ship Sanitation Control Exemption Certificate or a valid Ship Sanitation Control Certificate and outlines the necessary actions where these are not forthcoming.

The ID Aircraft Regulations set out the duties of the crew and commander of aircraft and those in charge of airports, and the powers and duties of health officers. In particular, the Aircraft Regulations set out the powers of the health officer in relation to the detention and inspection of aircraft and the inspection of passengers on board aircraft.

In 2009 an IHR Assessment Group Report made a number of recommendations in relation to preparedness in Ireland. A Medical Officer of Health (MOH) Port Health Committee was established as part of the response to this report. The terms of reference of this Committee, which consists of specialists in Public Health Medicine, are 1) to act as a forum for sharing experience and knowledge regarding preparedness for, and response to, communicable disease incidents at points of entry, and 2) to organise training and desktop exercises to test the guidelines that have been produced. In addition, the group also aims to progress multidisciplinary working in relation to Port Health.

### EVD and Port Health Preparedness in Ireland

In Ireland, EVD preparedness was coordinated nationally in the health service through the Department of Health and Ireland’s designated International Focal Point, the Health Protection Surveillance Centre (HPSC).

**Fig. 1:**
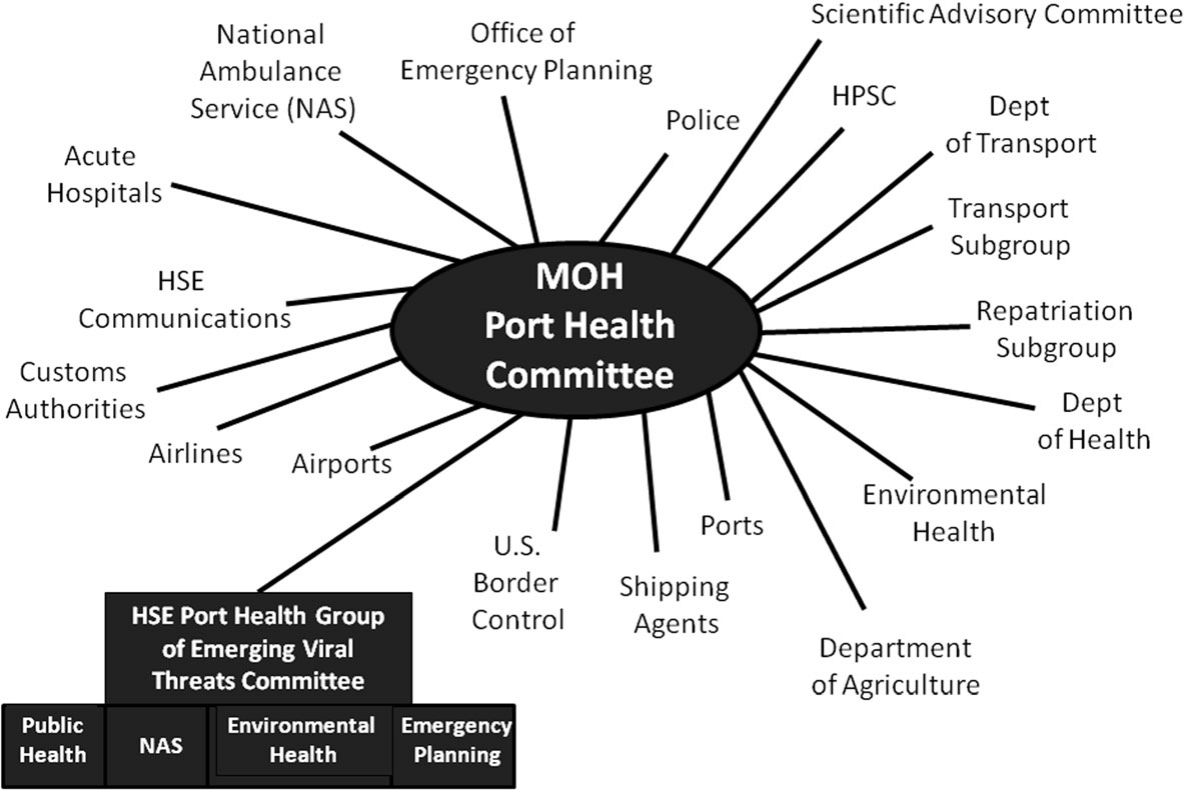
Collaboration between the Port Health Committee and other groups and organizations in Ireland

Following the declaration of the EVD outbreak as a Public Health Emergency of International Concern (PHEIC), the work of the Port Health Committee (hereafter referred to as ‘the Committee’) involved liaison with multiple other groups and organizations, including the HPSC, port and airport management, customs and immigration officials, the Office of Emergency Planning, Environmental Health and a number of government departments. During this period, the Committee also worked in collaboration with the health service’s Port Health Group, whose membership comprises Public and Environmental Health, the Department of Emergency Planning and the National Ambulance Service (NAS) (Fig. 1). The Committee aimed to improve knowledge and awareness for points of entry authorities (ports and airports), to assist in early detection of potentially infected persons, to disseminate health information for travelers, to develop protocols for assessment and case management, to ensure infection prevention and control at points of entry and to assist in implementing World Health Organisation (WHO) recommendations related to the management of EVD [4].

#### Actions taken – airlines and airports

**Fig. 2:**
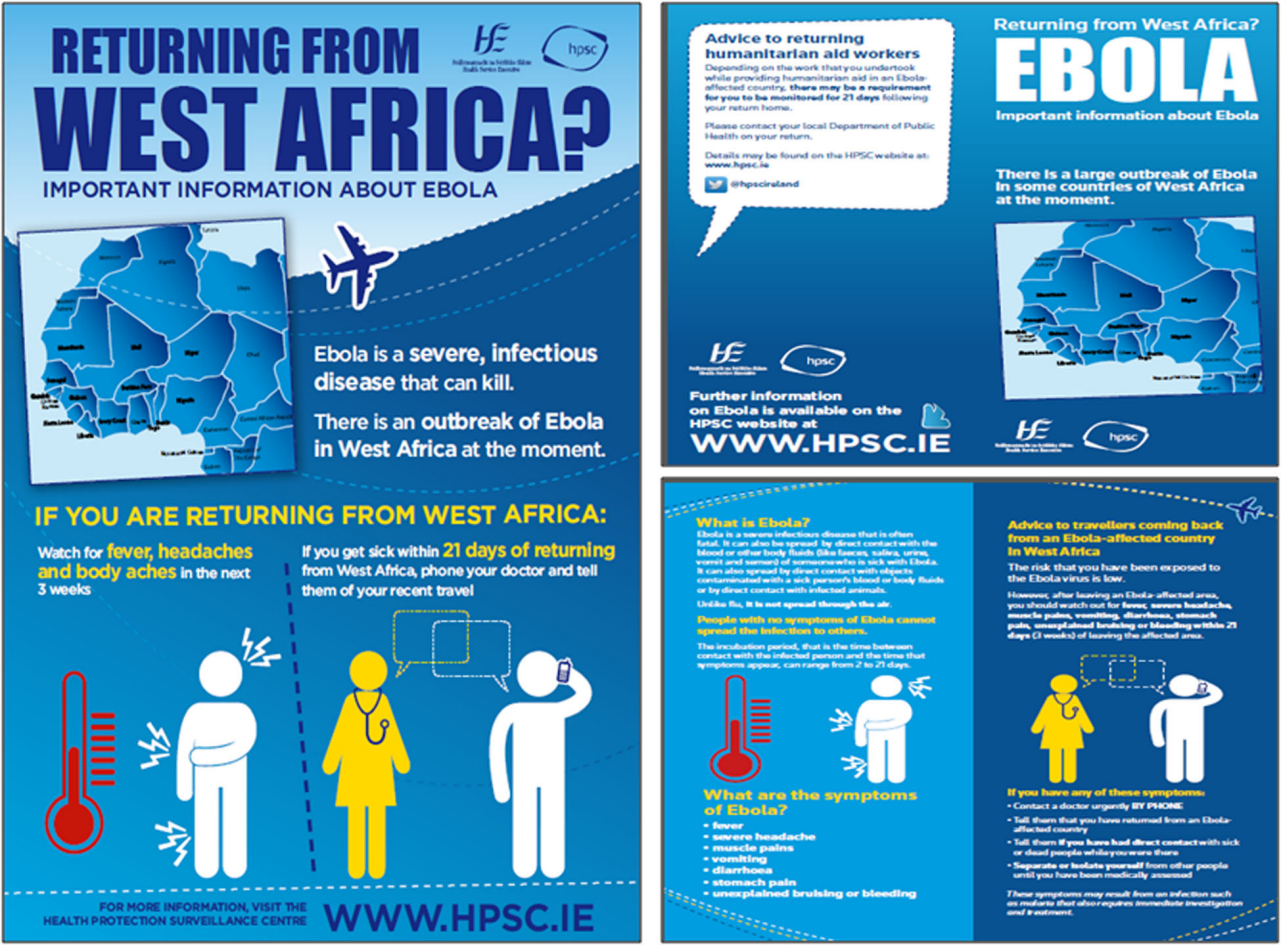
EVD Poster and Leaflet displayed in airports and ports in Ireland

The first information pack for airlines and airports was distributed to them on the 15th August 2014. This contained general advice about Ebola and the areas affected, advice for airline and cabin crew regarding necessary actions in the event of a suspected case on board a flight, as well as advice for cleaning and air cargo personnel. Some of the airports then sought additional advice for specific staff groups such as baggage handlers and immigration officers and presentations were prepared and given to the relevant stakeholders. In November 2014 25,000 information leaflets and posters in English, Irish and French were prepared and distributed through to Ireland’s six airports (Fig. 2). In February 2015 updated guidance was issued containing additional information for airport and airline staff in view of how the global situation had evolved over the preceding months.

Work was also done to progress general preparedness and strengthening at our airports. In January 2015, for example, the evidence regarding contact tracing of passengers on board a flight with a suspected case was reviewed in light of how this had been managed internationally. The guidance of international organizations including that of the European Centre for Disease Prevention and Control (ECDC) was reviewed and a position statement, in which it was proposed that only passengers who were one seat away from the index case should be traced back, was accepted by Ireland’s Scientific Advisory Committee.

Passenger Locator forms and on-board announcements for passengers were developed and airports were supplied with a stock of these forms. Contact tracing agreements were established with the NAS and the Committee contributed to the development of an updated management algorithm for members of that service. The period saw the development of relationships with Airport and Airline Authorities, a multidisciplinary desktop meeting was held in November 2014 and a practical repatriation exercise was carried out at Dublin Airport in January 2015.

#### Ports and shipping

A similar pattern of work was required in relation to Ireland’s shipping ports. Guidance was issued, this time in conjunction with the Environmental Health service, in September 2014. Presentations were given as required to specific groups including harbourmasters, and information leaflets and posters were distributed to 14 ports for display in terminals and on passenger ferries (Fig. 2). A protocol was agreed between Environmental Health and Public Health for the management of MDH from ships coming from affected areas, both in and out of hours. A practical exercise was held at Rusal Aughinish and again the period was used to develop and strengthen working relationships with all of the stakeholders involved.

## Discussion and conclusions

From a Public Health perspective, globalization means that a health threat in one country puts all at risk. In the case of EVD, people with infection crossed borders within Africa and to Europe and to North America where they unintentionally caused small chains of transmission far from the outbreak’s epicentre [5]. The actions taken globally in relation to EVD - and more recently Zika - have highlighted many of the inadequacies inherent within the international response mechanisms aimed at dealing with these crises, and health systems therefore need to be strengthened and prepared, not just for EVD and Zika, but for all new and emerging diseases which hold outbreak potential [6].

In Ireland, preparedness activities undertaken between August 2014 and March 2016 facilitated the development of cross-disciplinary working within the health service and forged relationships with external stakeholders including those at our ports and airports, and across government departments. Progress was also made at the operational level, with clarity brought to case and contact management through the development of agreed protocols. While welcome, this work also highlighted the extent to which preparedness requires ongoing and sustained commitment from all stakeholders, both nationally and internationally, in ensuring that countries are ready when the next threat presents at their borders.

## Abbreviations

ECDC, European Centre for Disease Prevention and Control; EVD, Ebola Virus Disease; HPSC, Health Protection Surveillance Centre; ID, infectious disease; IHR, International Health Regulations; MDH, Maritime Declaration of Health; MOH, Medical Officer of Health; NAS, National Ambulance Service; PHEIC, Public Health Emergency of International Concern; WHO, World Health Organisation
